# Efficacy and safety of rebamipide liquid for chemoradiotherapy-induced oral mucositis in patients with head and neck cancer: a multicenter, randomized, double-blind, placebo-controlled, parallel-group phase II study

**DOI:** 10.1186/s12885-017-3295-4

**Published:** 2017-05-05

**Authors:** T. Yokota, T. Ogawa, S. Takahashi, K. Okami, T. Fujii, K. Tanaka, S. Iwae, I. Ota, T. Ueda, N. Monden, K. Matsuura, H. Kojima, S. Ueda, K. Sasaki, Y. Fujimoto, Y. Hasegawa, T. Beppu, H. Nishimori, S. Hirano, Y. Naka, Y. Matsushima, M. Fujii, M. Tahara

**Affiliations:** 1 Division of Gastrointestinal Oncology, Shizuoka Cancer Center, 1007 Shimonagakubo, Nagaizumi-cho, Shizuoka, 411-8777 Japan; 2 Department of Otolaryngology-Head and Neck Surgery, Tohoku University Graduate School of Medicine, 2-1 Seiryo-machi, Aoba-ku, Sendai, Miyagi Japan; 3 Department of Medical Oncology, The Cancer Institute Hospital of JFCR, 3-8-31 Ariake, Koto-ku, Tokyo, 135-8550 Japan; 4 Department of Otolaryngology, Center of Head and Neck Surgery, Tokai University, 143 Shimokasuya, Isehara, Japan; 5 Department of Otolaryngology, Head and Neck Surgery, Osaka Medical Center for Cancer and Cardiovascular Diseases, Osaka, 537-8511 Japan; 6 Department of Medical Oncology, Kindai University Faculty of Medicine, Sayama, Osaka, 589-0014 Japan; 7 Department of Head and Neck Cancer, Hyogo Cancer Center, Akashi, 673-8558 Japan; 8 Department of Otolaryngology-Head and Neck Surgery, Nara Medical University, Kashiharashi, 634-8522 Japan; 9 Department of Otorhinolaryngology-Head and Neck Surgery, Hiroshima University Hospital, 1-2-3 Kasumi, Minami-ku, Hiroshima, 734-8551 Japan; 10 Department of Head and Neck Surgery, Shikoku Cancer Center, Matsuyama, 791-0280 Japan; 11 Department of Head and Neck Surgery, Miyagi Cancer Center, 47-1 Medeshimashiote, Natori, 981-1293 Japan; 12 Department of Otorhinolaryngology, Jikei University School of Medicine, 3-19 Nishi-Shinbashi, Minato-ku, Tokyo, 105-0003 Japan; 13 Medical Oncology, Nara Hospital, Kindai University School of Medicine, 1248−1 Otoda-cho, Ikoma, Nara, 630-0293 Japan; 14 Head and Neck, Chiba Cancer Center, 666-2 Nitona-cho, Chuo-ku, Chiba, 260-0801 Japan; 15 Department of Otorhinolaryngology, Nagoya University, Graduate School of Medicine, 65 Tsurumai-cho, Shouwa-ku, Nagoya, Aichi 466-8550 Japan; 16 Department of Head and Neck Surgery, Aichi Cancer Center Hospital and Research Institute, 1-1 Kanokoden, Chikusa-ku, Nagoya, 464-8681 Japan; 17 Division of Head and Neck Surgery, Saitama Cancer Center, 780 Komuro, Inamachi, Kitaadachi-gun, Saitama, Japan; 18 Department of Hematology and Oncology, Okayama University Hospital, 2-5-1 Shikata-cho, Kita-ku, Okayama, Japan; 19 Department of Otolaryngology-Head and Neck Surgery, Kyoto University Hospital, 54 Kawaharacho, Shogoin, Sakyo-ku, Kyoto, 606-8507 Japan; 20 Headquarters of New Product Evaluation and Development, Otsuka Pharmaceutical Co., Ltd., Shinagawa Grand Central Tower, 2-16-4 Konan, Minato-ku, Tokyo, 108-8242 Japan; 21 Department of Otolaryngology, Eiju General Hospital, 2-23-16 Higashiueno, Taito-ku, Tokyo, 110-8645 Japan; 22 Department of Head and Neck Medical Oncology, National Cancer Center Hospital East, 6-5-1 Kashiwanoha, Kashiwa, Chiba, 277-8577 Japan

**Keywords:** Chemoradiotherapy, Head and neck cancer, Oral mucositis, Rebamipide liquid, Randomized, Placebo-controlled

## Abstract

**Background:**

Recent preclinical and phase I studies have reported that rebamipide decreased the severity of chemoradiotherapy-induced oral mucositis in patients with oral cancer. This placebo-controlled randomized phase II study assessed the clinical benefit of rebamipide in reducing the incidence of severe chemoradiotherapy-induced oral mucositis in patients with head and neck cancer (HNC).

**Methods:**

Patients aged 20–75 years with HNC who were scheduled to receive chemoradiotherapy were enrolled. Patients were randomized to receive rebamipide 2% liquid, rebamipide 4% liquid, or placebo. The primary endpoint was the incidence of grade ≥ 3 oral mucositis determined by clinical examination and assessed by central review according to the Common Terminology Criteria of Adverse Events version 3.0. Secondary endpoints were the time to onset of grade ≥ 3 oral mucositis and the incidence of functional impairment (grade ≥ 3) based on the evaluation by the Oral Mucositis Evaluation Committee.

**Results:**

From April 2014 to August 2015, 97 patients with HNC were enrolled, of whom 94 received treatment. The incidence of grade ≥ 3 oral mucositis was 29% and 25% in the rebamipide 2% and 4% groups, respectively, compared with 39% in the placebo group. The proportion of patients who did not develop grade ≥ 3 oral mucositis by day 50 of treatment was 57.9% in the placebo group, whereas the proportion was 68.0% in the rebamipide 2% group and 71.3% in the rebamipide 4% group. The incidences of adverse events potentially related to the study drug were 16%, 26%, and 13% in the placebo, rebamipide 2%, and rebamipide 4% groups, respectively. There was no significant difference in treatment compliance among the groups.

**Conclusions:**

The present phase II study suggests that mouth washing with rebamipide may be effective and safe for patients with HNC receiving chemoradiotherapy, and 4% liquid is the optimal dose of rebamipide.

**Trial registration:**

ClinicalTrials.gov under the identifier NCT02085460 (the date of trial registration: March 11, 2014).

**Electronic supplementary material:**

The online version of this article (doi:10.1186/s12885-017-3295-4) contains supplementary material, which is available to authorized users.

## Background

Oral mucositis is an adverse event (AE) frequently induced by radiotherapy and chemotherapy during cancer treatment. Common symptoms are pain, dysphagia, dysgeusia, and infection, which can considerably affect the patient’s quality of life. Additionally, oral mucositis is a risk factor for sepsis in patients with low neutrophil count secondary to cancer treatment toxicity. During cancer treatment, aggravation of oral mucositis leads to dose reduction, suspension, or discontinuation of treatment, thereby affecting the patient prognosis [1, 2].

Approximately 600,000 patients worldwide are currently undergoing cancer treatment with radiotherapy and/or chemotherapy, and are at risk of developing oral mucositis [3]. Reportedly, oral mucositis develops in more than 90% of patients receiving chemoradiotherapy (CRT) for head and neck cancer (HNC) [4]. Thus, there is a particularly strong demand for the development of prophylactic and therapeutic agents for oral mucositis.

Oral mucositis results from direct cell injury caused by chemotherapy or radiotherapy. Tissue injury is amplified by reactive oxygen species, proinflammatory cytokines and pathways, and metabolic byproducts of colonizing microorganisms [1]. Some agents targeting these underlying mechanisms have been evaluated for oral mucositis treatment. Palifermin, a keratinocyte growth factor-1, has been approved by the Food and Drug Administration for stem cell transplantation; however, it has not been approved for HNC, although it was shown to decrease oral mucositis effectively [5, 6]. Rebamipide, originally developed by Otsuka Pharmaceutical Co., Ltd. (Tokyo, Japan) for gastritis, gastric ulcer, and xerophthalmia enhances endogenous prostaglandin production in the gastric mucosa and inhibits free radical production [7–9]. Additionally, it has been shown to inhibit neutrophil activation and inflammatory cytokine production by mononuclear cells, gastric mucosa, and vascular endothelial cells, and to inhibit other inflammatory reactions [10–12].

In pilot studies performed to assess the efficacy of rebamipide liquid for CRT-induced oral mucositis in patients with oral cancer, rebamipide decreased the severity of mucositis [13, 14]. Additionally, rebamipide liquid administered at doses of 1%, 2%, and 4%, resulted in a dose-dependent reduction of total injury extension and tongue ulcerations in a rat model of irradiation-induced oral mucositis [15].

In accordance with these preclinical and phase I studies, rebamipide 2% and 4% liquids were chosen as potential prophylactic and therapeutic agents for CRT-induced oral mucositis in patients with HNC. The aim of this phase II exploratory study was to compare the incidence of oral mucositis in patients receiving rebamipide 2% and 4% liquids, or placebo.

## Methods

### Study design

This was a multicenter, randomized, double-blind, placebo-controlled, parallel-group, dose-ranging phase II study. This study is registered at ClinicalTrials.gov under the identifier NCT02085460 (the date of trial registration: March 11, 2014). The institutional review boards of the 20 participating institutions (Additional file 1) approved the study protocol. All study procedures were conducted in accordance with the 1964 Declaration of Helsinki and its later amendments, and in compliance with the Good Clinical Practice Guidelines specified by the Ministry of Health, Labour and Welfare of Japan.

Dynamic allocation was used to randomize patients, with stratification based on the purpose of CRT for HNC (definitive or post-operative) and primary site (oral cavity, nasopharynx, oropharynx, hypopharynx, or larynx). Subject enrollment and random study drug allocation were performed using the Interactive Web Response System. The subjects and investigators were kept masked to the treatment allocation until the end of the study. A sample size of 90 subjects was planned based on the feasibility of patient enrollment. Patients were randomly assigned to one of three groups, with 30 patients each: placebo, rebamipide 2% liquid, and rebamipide 4% liquid.

### Patients

All study participants provided written informed consent at enrollment, 10 to 28 days prior to the initiation of CRT. Screening was performed based on the inclusion and exclusion criteria (Additional file 1). Briefly, patients between the ages of 20 and 75 years with histopathological diagnosis of primary tumor in the nasopharynx, oropharynx, hypopharynx, larynx, or oral cavity, regardless of the stage, scheduled to undergo definitive or postoperative CRT with ≥50 Gy irradiation to the buccal mucosa, floor of the mouth, tongue, or soft palate, with an Eastern Cooperative Oncology Group (ECOG) performance status (PS) score of 0 or 1, life expectancy of at least 3 months, without a history of chemotherapy, radiotherapy, or CRT for HNC, who could perform mouth washing and swallow fluids, and could attend follow-up visits, were included in this study.

### Study treatment

The study drugs consisted of placebo (same formulation as rebamipide liquids) and rebamipide 2% and 4% liquids, which were given 6 times daily, preferably once after every meal, twice between meals, and once before bedtime. Patients were instructed to wash their mouth with 5 mL of the study drug for at least 30 s and then swallow it. The use of all other drugs for oral mucositis treatment, including oral ointment of corticosteroid, was prohibited. The only local treatments permitted in this study were oral aerosol spray for xerostomia, local antibiotics for the treatment of infection (e.g., amphotericin b or miconazole), and analgesic agents (e.g., xylocaine, opioid analgesics, and acetaminophen).

The treatment started 3 days prior to the initiation of CRT and continued for another 77 days. Cisplatin at 80–100 mg/m^2^ was administered thrice at 3-week intervals. Radiotherapy at ≤2.2 Gy/fraction was administered once daily, with 5 fractions per week, up to a total dose of ≥60 Gy. Withdrawal from the study was accepted before Day 77 if oral mucositis resolved completely, if oral mucositis did not develop 1 week after CRT completion, or if a patient requested to withdraw from the study.

Post-treatment examinations, including the assessment of adverse events and observation of oral mucositis, were performed 1 week after completion of CRT or 1 week after the patient decided to withdraw from the study. Subjects who underwent post-treatment examination were defined as subjects who completed the study.

### Oral assessment

Investigators who had undergone specific training assessed the severity of oral mucositis twice every week. To evaluate the severity of oral mucositis objectively, the clinical findings of the oral mucosa as well as functional disorders and symptomatic aspects were recorded in the Oral Mucositis Assessment Sheet (Additional file 1) by each investigator. Photographic documentation of the oral mucosa was also submitted by each investigator, 3 days before or 57 days after initiation of CRT, or at the time of withdrawal. The Oral Mucositis Assessment Sheet allows the relevant findings at each site in the oral cavity (10 sites in total) to be recorded separately. The Oral Mucositis Assessment Sheets and photographic documentation were then reviewed by the Oral Mucositis Evaluation Committee to grade the severity of oral mucositis according to the Common Terminology Criteria for Adverse Events (CTCAE) 3.0. Grade 3 for clinical examination was defined as “confluent ulcerations or pseudomembranes (bleeding with minor trauma),” and grade 3 for function/symptoms was defined as “symptomatic and unable to adequately ingest food or hydrate orally.”

### Endpoints

The primary endpoint was the incidence of grade ≥ 3 oral mucositis assessed via clinical examination according to the CTCAE version 3.0. Secondary endpoints were time to onset of grade ≥ 3 oral mucositis and the incidence of functional impairment (grade ≥ 3) based on the Oral Mucositis Evaluation Committee evaluation. Exploratory endpoints were total cisplatin dose and compliance with radiotherapy during the study. Pharmacokinetics was assessed in terms of safety in the patients who ingested the drug. The AEs were assessed and classified based on the MedDRA system organ class and preferred term.

### Statistical analysis

The full analysis set (FAS) comprised patients who received the study drug or placebo at least once and whose efficacy data were collected immediately after beginning the treatment. The safety set (SS) comprised those who received the study drug or placebo at least once and whose safety data were collected at least once after beginning the treatment. The per protocol set (PPS) consisted of patients who were compliant with the protocol. Patients who did not satisfy the inclusion/exclusion criteria, did not receive adequate radiotherapy, or did not comply with the prescription of combination therapy, or show drug compliance were excluded from the PPS.

The incidence of oral mucositis in each group was compared using the chi-square test. A step-down strategy was used for the between-group comparison, adjusting for multiplicity. Comparisons were made first between the rebamipide 4% and the placebo groups, and then between the rebamipide 2% and placebo groups. The Cochran-Armitage test was used as a trend test. All statistical analyses were performed using SAS 9.2 (SAS Institute Japan, Tokyo, Japan).

## Results

### Patient characteristics

Of 97 subjects randomized between April 2014 and August 2015, approximately 50% of the subjects had a primary tumor in the oropharynx, and approximately 20% of the subjects had a history of surgery for head and neck cancer. However, as a whole, the baseline characteristics of patients were well balanced between the treatment groups (Table 1).

**Table 1:** Patient baseline characteristics

	Placebo (*N* = 31)	Rebamipide 2% (*N* = 31)	Rebamipide 4% (*N* = 32)
Male (%)	25 (81)	26 (84)	26 (81)
Age (mean ± SD) (%)	60 ± 9	61 ± 12	62 ± 9
ECOG PS (PS = 0) (%)	28 (90)	28 (90)	28 (88)
Primary site			
Oral cavity (%)	2 (6)	4 (13)	4 (13)
Nasopharynx (%)	6 (19)	7 (23)	6 (19)
Oropharynx (%)	17 (55)	14 (45)	15 (47)
Hypopharynx (%)	5 (16)	6 (19)	6 (19)
Larynx (%)	1 (3)	0	1 (3)
Prior surgery for head and neck cancer (with prior surgery) (%)	7 (23)	7 (23)	6 (19)
TNM staging of primary tumor			
T1	6 (19)	9 (29)	4 (13)
T2	14 (45)	10 (32)	15 (47)
T3	7 (23)	4 (13)	5 (16)
T4	4 (13)	8 (26)	8 (25)
N0	7 (23)	3 (10)	3 (9)
N1	8 (26)	3 (10)	8 (25)
N2	13 (42)	23 (74)	20 (63)
N3	3 (10)	2 (6)	1 (3)
Radiation technique			
3D–CRT	4 (13)	9 (29)	7 (22)
IMRT	27 (87)	22 (71)	25 (78)

Data are presented as number and percent [n (%)]

*SD* standard deviation, *ECOG PS* Eastern Cooperative Oncology Group performance status, *3D–CRT* three-dimensional conformal radiation therapy, *IMRT* Intensity-Modulated Radiation Therapy

### Patient disposition

**Fig. 1:**
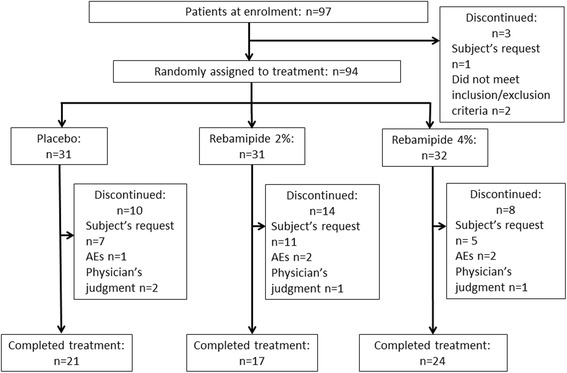
Patient disposition by individual treatment group. AE, adverse event

A total of 94 patients received the study drug and were included in the FAS and the SS. Sixty-two (66%) patients completed the study. The most frequent reason for study withdrawal in all three groups was patient request (22%, 33%, and 16% in the placebo, rebamipide 2% and 4% groups, respectively) (Fig. 1).

### Incidence of oral mucositis

**Fig. 2:**
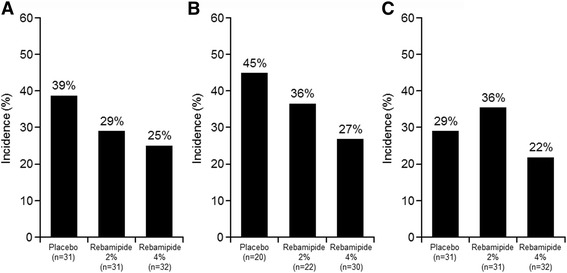
Incidence of grade ≥ 3 oral mucositis based on clinical examination by full analysis set (FAS) (**a**) and by per protocol set (PPS) (**b**); **c** Incidence of functional impairment (grade ≥ 3)

In the FAS, the incidence of grade ≥ 3 oral mucositis determined by clinical examination and assessed by the Oral Mucositis Evaluation Committee was 29% and 25% in the rebamipide 2% and 4% groups, respectively, compared with 39% in the placebo group (Fig. 2a). In a trend test, a decrease in the incidence of grade ≥ 3 oral mucositis was observed with an increasing concentration of rebamipide liquid; however, this decrease was not statistically significant (*p* = 0.2399). In the PPS, the incidence of grade ≥ 3 oral mucositis was 45% (*n* = 20), 36% (*n* = 22), and 27% (*n* = 30) in the placebo, rebamipide 2%, and rebamipide 4% groups, respectively, with no significant difference (*p* = 0.1779) (Fig. 2b). The incidence of functional impairment (Grade 3 or higher) was 29%, 36%, and 22% in the placebo, rebamipide 2% and 4% groups, respectively (Fig. 2c).

### Time to onset of grade ≥ 3 oral mucositis

**Fig. 3:**
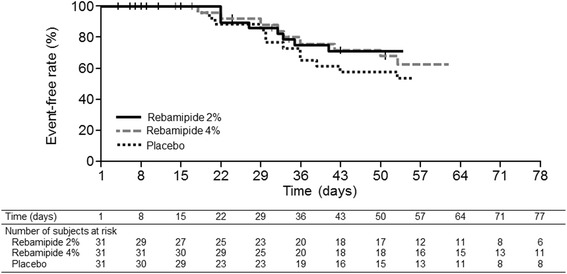
Time to onset of grade ≥ 3 oral mucositis. The y-axis shows the percentage of patients who have not developed grade ≥ 3 mucositis

The rebamipide 2% and 4% groups showed a trend of delaying the time to onset of grade ≥ 3 oral mucositis as compared with the placebo group, although the difference between the groups was not statistically significant (Fig. 3). For instance, the proportion of patients who did not develop grade ≥ 3 oral mucositis by day 50 of treatment was 57.9% in the placebo group, whereas the proportion was 68.0% in the rebamipide 2% group and 71.3% in the rebamipide 4% group.

### Treatment compliance

Oral retention and swallowing compliance for the study drugs were better in the rebamipide groups than in the placebo group. The proportion of patients whose oral retention and swallowing compliance was ≥80% was highest in the rebamipide 4% group, being 78.1%. In contrast, the proportion in the rebamipide 2% group was the same as that in the placebo group (58.1%). No significant differences in the total doses of cisplatin and the total radiation dose were observed among the groups (Table 2), suggesting that compliance with CRT was not influenced by the study drug.

**Table 2:** Treatment compliance

Number of doses	Placebo *N* = 31	Rebamipide 2% *N* = 31	Rebamipide 4% *N* = 32
	297 ± 168	278 ± 171	343 ± 135
Retention compliance,^a^ n (%)			
≥ 80%	18 (58.1)	18 (58.1)	25 (78.1)
≥ 50% to <80%	6 (19.4)	7 (22.6)	6 (18.8)
< 50%	7 (22.6)	6 (19.4)	1 (3.1)
Swallowing rate,^b^ n (%)			
≥ 80%	9 (29.0)	13 (41.9)	14 (43.8)
≥ 50% to <80%	6 (19.4)	8 (25.8)	6 (18.8)
< 50%	16 (51.6)	10 (32.3)	12 (37.5)
Total doses of cisplatin (mg/m^2^)	213 ± 73	192 ± 66	233 ± 61
The total radiation dose (Gy)	58 ± 20	55 ± 22	63 ± 13
Frequency of interruption of radiotherapy			
0 times	28 (90.3)	29 (93.5)	30 (93.8)
1 times	3 (9.7)	2 (6.5)	1 (3.1)
2 times	0	0	0
3 times	0	0	0

Number of doses, total doses of cisplatin, and total radiation dose are expressed as mean ± standard deviation

^a^Retention means keeping the investigational medicinal product for 30 s or more in the mouth. Retention compliance (%) = Total number of investigational medicinal product (IMP) retention ÷ [(end date of IMP administration - start date of IMP administration +1) × 6] × 100

^b^Swallowing rate (%) = Total number of IMP swallowed ÷ [(end date of IMP administration - start date of IMP administration +1) × 6] × 100

### Pharmacokinetics

Peak plasma concentration (mean ± standard deviation) of rebamipide on day 64 was 241 ± 160 ng/mL in the rebamipide 2% group (*n* = 11) and 568 ± 235 ng/mL in the rebamipide 4% group (*n* = 15) (Additional file 2: Fig. S1). No remarkable inter-patient variation was observed. The plasma concentration of rebamipide did not reach a sufficient level to induce the biochemical effects of rebamipide.

### Safety

The incidence of AEs potentially related to the study drug was 16% (5/31), 26% (8/31), and 13% (4/32) in the placebo, rebamipide 2%, and rebamipide 4% groups, respectively. Nausea and vomiting were the most frequently reported AEs (Table 3). All patients experienced at least one AE and there was no significant difference in the incidence among the groups (data not shown).

**Table 3:** Incidence of potentially drug-related treatment-emergent adverse events (TEAEs) - Safety Analysis Set

	Placebo *N* = 31 n (%)	Rebamipide 2% *N* = 31 n (%)	Rebamipide 4% *N* = 32 n (%)
Total number of subjects with TEAEs	5 (16)	8 (26)	4 (13)
Nausea	2 (7)	2 (7)	0
Vomiting	0	3 (10)	0
Constipation	0	1 (3)	0
Stomatitis	1 (3)	0	0
Hepatic function abnormal	1 (3)	1 (3)	1 (3)
Oral fungal infection	1 (3)	0	0
Blood creatinine increased	0	1 (3)	1 (3)
Gamma-glutamyltransferase increased	0	1 (3)	1 (3)
Blood alkaline phosphatase increased	0	0	1 (3)
Decreased appetite	0	1 (3)	0
Irritability	1 (3)	0	1 (3)
Renal impairment	0	1 (3)	0
Rash	0	1 (3)	0

## Discussion

Basic oral care is considered common sense in the management of radiation-induced mucositis. However, a systematic oral care program alone is insufficient to decrease the incidence of severe oral mucositis in patients with HNC undergoing CRT [16]. Therefore, there is a strong demand for the development of prophylactic and therapeutic agents against oral mucositis. This phase II study evaluated the suppressive effect and safety of rebamipide liquid for CRT-induced oral mucositis in patients with HNC and assessed the optimal dose of rebamipide liquid.

As reported by the investigators and evaluated by the Oral Mucositis Evaluation Committee, we observed a decreased incidence of grade ≥ 3 oral mucositis in patients treated with rebamipide 2% and 4% liquids compared with those treated with placebo; however, these differences were not statistically significant. Furthermore, there was a trend towards a prolongation in the time to onset of grade ≥ 3 oral mucositis and a decrease in functional impairment in patients treated with rebamipide 4% liquid compared with those treated with placebo. These results may suggest a clinical benefit of rebamipide in reducing the incidence of severe oral mucositis induced by CRT. Recently, a small randomized, double-blind, placebo-controlled trial was conducted in patients with oral cancer treated with CRT at a total radiation dose of 40 Gy and concomitant weekly chemotherapy with docetaxel 10 mg/m^2^. The results revealed that the incidence of grade ≥ 3 mucositis (World Health Organization grade 3 or 4) in patients receiving rebamipide 0.1% was 33% (*p* = 0.036), compared with 83% in patients receiving placebo [9]. Even though the mean total radiation dose in our study was higher than that in their study, our patients had a lower incidence of grade ≥ 3 mucositis with rebamipide 4% liquid.

Previous reports showed that a rebamipide concentration ≥ 10 μM was required to exhibit its inhibitory effect on the production of radical and inflammatory cytokines [8, 9, 11, 12]. In this study, the plasma concentration in the rebamipide 4% group was 568 ± 235 ng/mL (1.53 μM). This suggests that the concentration reached after swallowing rebamipide, and its subsequent intestinal absorption, was insufficient to achieve a biologically active plasma concentration. We hypothesize that the local concentration of rebamipide achieved through mouth washing plays a major role in the clinical effect of rebamipide on oral mucositis. Furthermore, in patients who underwent definitive CRT, the response rate was 62.5%, 62.5%, and 69.2% in the placebo, rebamipide 2% and 4% groups, respectively. No new lesions were observed in all patients who underwent postoperative CRT at the point of the first scan. These results may suggest that rebamipide has no remarkable effect on local disease control despite its free radical scavenging effect on reactive oxygen species.

Although all patients reported at least one AE, there were no significant differences in the incidence of AEs among the groups. Therefore, no concerns were raised regarding the safety profiles of either rebamipide 2% or rebamipide 4% liquid. The safety profile of rebamipide suggests that ingestion of rebamipide 2% and 4% liquid formulations is safe.

The efficacy and safety profiles suggest that the optimal dose of rebamipide for the next phase of the study is rebamipide 4% liquid. Unfortunately, no statistically significant differences were observed between the rebamipide liquids and placebo with regard to the suppressive effect on CRT-induced oral mucositis. Several factors may explain this negative result. The first is the poor compliance in the rebamipide 2% and 4% groups, and the placebo group. The most common reason for discontinuation of treatment was related with the taste and smell of the formulation. For instance, in a recent patient survey of Japanese patients with liver cirrhosis, poor adherence to treatment with branched-chain amino acid granules was significantly associated with disinterest and distaste owing to the flavor and volume of the medication [17]. Their finding and ours suggest that patients are highly susceptible to the smell and taste of medications that require frequent intake. Therefore, the taste of the medication needs to be improved in the future. The second factor is the small sample size. A small sample size and poor compliance with the study drug may lead to statistically underpowered results. Further, in the present study, subjects who discontinued treatment were not followed-up after the treatment discontinuation. Therefore, there is a possibility that compliance with CRT and the incidence of grade ≥ 3 oral mucositis were not assessed accurately in the subjects who discontinued the treatment. Because of these factors and the lack of statistical significance, the benefit of rebamipide might be underestimated.

There were almost no differences in functional and symptomatic aspects between the placebo and rebamipide groups. The functional and symptomatic aspects are not only affected by mucositis, but also by a combination of multiple factors, including dysgeusia, salivary gland secretion, and swallowing dysfunction associated with irradiation of the pharyngeal constrictor muscles. Therefore, control of mucositis only by rebamipide may not be sufficient to prevent functional impairment.

## Conclusions

Our study indicates that mouth washing with rebamipide liquid may be potentially effective and safe for patients with HNC receiving CRT. The efficacy and safety profiles suggest that 4% liquid is the optimal dose of rebamipide. Based on the present results and those of previous pilot studies [13, 14], we consider that it is highly relevant to conduct the next phase of study with a larger sample size.

## Additional files

Additional file 1: Supplementary Information: centers participating in the study; inclusion and exclusion criteria; oral mucositis assessment sheet. (DOCX 25 kb)

Additional file 2: Fig. S1. Mean peak plasma concentrations of rebamipide. (DOCX 37 kb)

## Abbreviations

AE: Adverse event
CRT: Chemoradiotherapy
CTCAE: Common Terminology Criteria for Adverse Events
ECOG: Eastern Cooperative Oncology Group
FAS: Full analysis set
HNC: Head and neck cancer
PPS: Per protocol set
PS: Performance status
