# Supplementary material for: Efficacy and safety of rebamipide liquid for chemoradiotherapy-induced oral mucositis in patients with head and neck cancer: a multicenter, randomized, double-blind, placebo-controlled, parallel-group phase II study
Source: BMC Cancer. 2017 May 5;17:314. doi: 10.1186/s12885-017-3295-4 (PMC5420134; doi:10.1186/s12885-017-3295-4)
Supplement: Supplementary file 1 — Supplementary Information: centers participating in the study; inclusion and exclusion criteria; oral mucositis assessment sheet. (DOCX 25 kb) [file 12885_2017_3295_MOESM1_ESM.docx]

**Additional file 1**

**Centers that participated in this study**

Tohoku University Hospital, The Cancer Institute Hospital Of JFCR, Tokai University Hospital, Shizuoka Cancer Center, Osaka Medical Center for Cancer and Cardiovascular Diseases, Kindai University Hospital, Hyogo Cancer Center, Nara Medical University Hospital, Hiroshima University Hospital, National Hospital Organization Shikoku Cancer Center, Miyagi Cancer Center, The Jikei University Hospital, Kindai University, Nara Hospital, Chiba Cancer Center, Nagoya University Hospital, Aichi Cancer Center Hospital, Saitama Cancer Center, Okayama University Hospital, Kyoto University Hospital.

**Inclusion and exclusion criteria**

Inclusion criteria:

1) Patients with head and neck cancer scheduled to undergo definitive or postoperative chemoradiotherapy with ≥50 Gy irradiation to at least one of the following: buccal mucosa, floor of the mouth, tongue, or soft palate.

2) Patients with a histopathological diagnosis of head and neck cancer and primary tumor in one of the following regions:

a) Definitive therapy: nasopharynx, oropharynx, hypopharynx, or larynx

b) Postoperative treatment: oral cavity, oropharynx, hypopharynx, or larynx

3) Patients with no history of chemotherapy, radiotherapy, or chemoradiotherapy for head and neck cancer

4) Patients aged 20 to ≤75 years at the time of providing informed consent

5) Patients with an ECOG performance status score of 0 or 1

6) Patients who are able to hold fluid in the mouth

7) Patients who are able to swallow the study drug

8) Patients expected to survive for at least 3 months

9) Patients who have given written informed consent in person

10) Patients who can stay at or visit the hospital for scheduled examinations and observations

11) Patients who are able to take contraceptive measures to avoid pregnancy or pregnancy in their partner from the time of informed consent until 4 weeks after completion of study drug administration

Exclusion criteria:

1) Patients with primary malignant tumors other than head and neck cancer (except for patients with endoscopically resectable early gastrointestinal cancer and patients with no recurrence of other cancers for at least 3 years).

2) Patients with symptomatic viral, bacterial, or fungal infection (except for tinea pedis)

3) Patients with serious renal impairment

4) Patients with distant metastasis

5) Patients with severe complications (uncontrolled cardiac disease, diabetes, hypertension, etc.) that would make study participation difficult

6) Patients with any of the following laboratory test results:

a) Neutrophil count: <1500/μL

b) Platelet count: <75 000/μL

c) Hemoglobin: <10.0 g/dL

d) AST: >3 times the upper limit of the reference value at the study site.

e) ALT: >3 times the upper limit of the reference value at the study site.

f) Serum bilirubin: >1.5 times the upper limit of the reference value at the study site.

g) Serum albumin: <3.0 g/dL

h) Serum creatinine: >1.5 times the upper limit of the reference value at the study site.

i) Creatinine clearance (Ccr): <30 mL/min (Ccr calculated by the Cockcroft-Gault formula)

7) Patients with autoimmune disease (rheumatoid arthritis, systemic lupus erythematosus, connective tissue disease, Behçet’s disease, etc.)

8) Patients requiring continuous systemic administration of glucocorticoid

9) Female patients who are pregnant or lactating, who may possibly be pregnant, or who wish to become pregnant

10) Patients who have participated in any other clinical trial within 4 weeks prior to initiation of chemoradiotherapy

11) Patients who have a history of drug allergy to rebamipide, cisplatin, or other platinum compounds

12) Patients who were otherwise judged by the investigator or sub-investigator to be inappropriate for inclusion in the trial

**Oral mucositis assessment sheet**

| Subject No.: | - |
| --- | --- |
| Date of assessment: | Month/date/year |
| Assessor: |  |

| Observation | | | No change in the mucosa | Redness (erythema) | Color changes in the mucosa to white or pallor | Mucosal edema | Detachment of the epithelium from small ulcerative lesions (sporadic and non-contiguous) | White pseudomembrane covering the surface | Widespread ulcerative lesions over subsites by >30 mm | Easy bleeding from ulcerative lesions with contact | Spontaneous bleeding from ulcerative lesions | Necrosis of ulcerative surface | Mucosa not evaluable (flaps, etc.) |
| --- | --- | --- | --- | --- | --- | --- | --- | --- | --- | --- | --- | --- | --- |
|  |  |  | 0 | 1 | 2 | 3 | 4 | 5 | 6 | 7 | 8 | 9 | 88 |
| Lips | Upper lip | A1 | □ | □ | □ | □ | □ | □ | □ | □ | □ | □ | □ |
|  | Lower lip | A2 | □ | □ | □ | □ | □ | □ | □ | □ | □ | □ | □ |
| Buccal mucosa | Right side | B1 | □ | □ | □ | □ | □ | □ | □ | □ | □ | □ | □ |
|  | Left side | B2 | □ | □ | □ | □ | □ | □ | □ | □ | □ | □ | □ |
| Tongue | Dorsum of tongue | C1 | □ | □ | □ | □ | □ | □ | □ | □ | □ | □ | □ |
|  | Right lateral tongue | C2 | □ | □ | □ | □ | □ | □ | □ | □ | □ | □ | □ |
|  | Left lateral tongue | C3 | □ | □ | □ | □ | □ | □ | □ | □ | □ | □ | □ |
|  | Back of tongue- floor of the mouth | C4 | □ | □ | □ | □ | □ | □ | □ | □ | □ | □ | □ |
| Palate | Hard palate | D1 | □ | □ | □ | □ | □ | □ | □ | □ | □ | □ | □ |
|  | Soft palate- arch of palate | D2 | □ | □ | □ | □ | □ | □ | □ | □ | □ | □ | □ |

| Oral intake status | E | □^0^ Good oral intake: normal food (the same texture as that for the family) intake is maintained. |
| --- | --- | --- |
|  |  | □^1^ Oral intake is feasible with slight modifications in seasoning and texture of food (soft food, etc.) |
|  |  | □ The proportion of rice porridge, liquid diet and dietary supplements is ≥50%. |
|  |  | □^2^ The main causes are nausea and dysgeusia. |
|  |  | □^3^ The main causes are pain in the oral cavity (mucositis, etc.). |
|  |  | □ Inability to adequately aliment orally (gastric fistula, tube feeding) |
|  |  | □^4^ The main causes are nausea and dysgeusia |
|  |  | □^5^ The main causes are pain in the oral cavity (mucositis, etc.). |

*Check boxes that apply.
