# Supplementary material for: Efficacy and safety of rebamipide liquid for chemoradiotherapy-induced oral mucositis in patients with head and neck cancer: a multicenter, randomized, double-blind, placebo-controlled, parallel-group phase II study
Source: BMC Cancer. 2017 May 5;17:314. doi: 10.1186/s12885-017-3295-4 (PMC5420134; doi:10.1186/s12885-017-3295-4)

**Additional file 2**

**Figure S1.** Mean plasma concentrations of rebamipide. The error bars show the standard deviations.


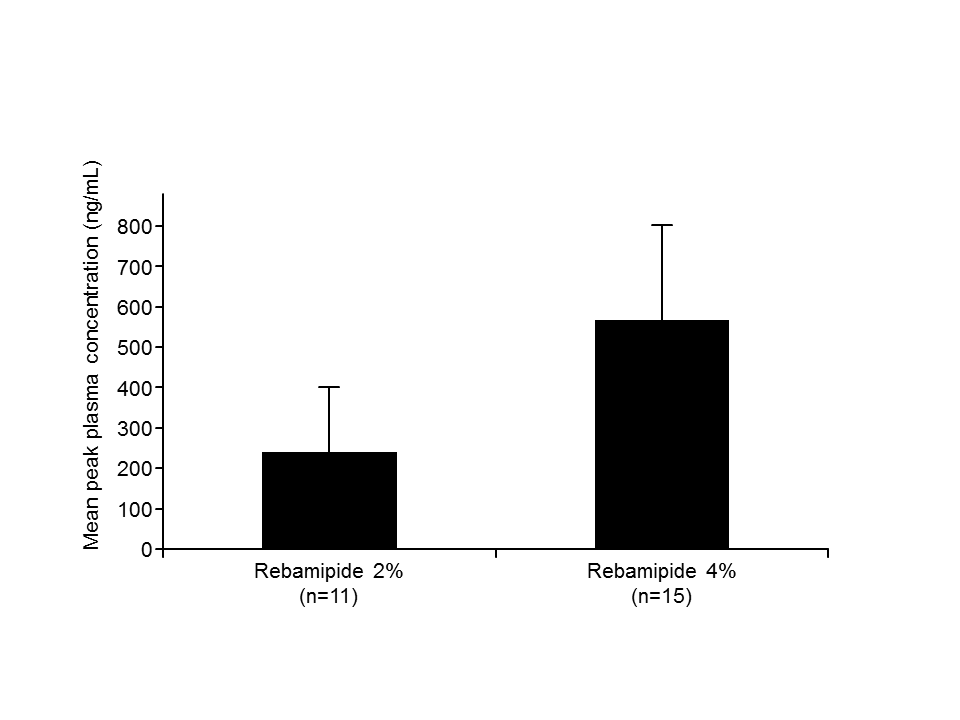

Supplement: Supplementary file 2 — Mean peak plasma concentrations of rebamipide. (DOCX 37 kb) [file 12885_2017_3295_MOESM2_ESM.docx]
